# Feasibility and accuracy of noninvasive continuous hemoglobin monitoring using transesophageal photoplethysmography in porcine model

**DOI:** 10.1186/s12871-024-02435-7

**Published:** 2024-02-06

**Authors:** Ling Peng, Long Zhao, Xue Zhang, Yi Zhang, Meng Ding, Zhibin Lin, Hao Jiang, Yuchen Huang, Bo Gao, Wei Wei

**Affiliations:** 1 Department of Anesthesiology, West China Hospital, Sichuan University, 37 Guo Xue Xiang, Chengdu, 610041 China; 2 Department of Cardiovascular Surgery, The Third People’s Hospital of Chengdu, 82 Qing Long Xiang, Chengdu, 610041 China; 3 Department of Physics, Sichuan University, Chengdu, 610064 China

**Keywords:** Photoplethysmography, Hemoglobin, Descending aorta, Near-infrared spectroscopy, Monitoring

## Abstract

**Background:**

Continuous and noninvasive hemoglobin (Hb) monitoring during surgery is essential for anesthesiologists to make transfusions decisions. The aim of this study was to investigate the feasibility and accuracy of noninvasive and continuous Hb monitoring using transesophageal descending aortic photoplethysmography (dPPG) in porcine model.

**Methods:**

Nineteen landrace pigs, aged 3 to 5 months and weighing 30 to 50 kg, were enrolled in this study. A homemade oximetry sensor, including red (660 nm) and infrared (940 nm) lights, was placed in the esophagus for dPPG signal detection to pair with the corresponding reference Hb values (Hb_i−STAT_) measured by blood gas analysis. The decrease and increase changes in Hb concentration were achieved by hemodilution and transfusion. Metrics, including alternating current (AC), direct current (DC), and AC/DC for both red and infrared light were extracted from the dPPG signal. A receiver operating characteristic (ROC) curve was built to evaluate the performance of dPPG metrics in predicting the Hb “trigger threshold” of transfusion (Hb < 60 g/L and Hb > 100 g/L). Agreement and trending ability between Hb measured by dPPG (Hb_dPPG_) and by blood gas analysis were analyzed by Bland-Altman method and polar plot graph. Error grid analysis was also performed to evaluate clinical significance of Hb_dPPG_ measurement.

**Results:**

The dPPG signal was successfully detected in all of the enrolled experimental pigs, without the occurrence of a continuous loss of dPPG signal for 2 min during the entire measurement. A total of 376 pairs of dPPG signal and Hb_i−STAT_ were acquired. AC_red_/DC_red_ and AC_inf_/DC_inf_ had moderate correlations with Hb_i−STAT_, and the correlation coefficients were 0.790 and 0.782, respectively. The areas under the ROC curve for AC_red_/DC_red_ and AC_inf_/DC_inf_ in predicting Hb_i−STAT_ < 60 g/L were 0.85 and 0.75, in predicting Hb_i−STAT_ > 100 g/L were 0.90 and 0.83, respectively. Bland-Altman analysis and polar plot showed a small bias (1.69 g/L) but a wide limit of agreement (-26.02–29.40 g/L) and a poor trend ability between Hb_dPPG_ and Hb_i−STAT_. Clinical significance analysis showed that 82% of the data lay within the Zone A, 18% within the Zone B, and 0% within the Zone C.

**Conclusion:**

It is feasible to establish a noninvasive and continuous Hb monitoring by transesophageal dPPG signal. The AC_red_/DC_red_ extracted from the dPPG signal could provide a sensitive prediction of the Hb threshold for transfusion. The Hb concentration measured by dPPG signal has a moderate correlation with that measured by blood gas analysis. This animal study may provide an experimental basis for the development of bedside Hb_dPPG_ monitoring in the future.

## Background

Reliable and continuous noninvasive hemoglobin (Hb) monitoring is essential for anesthesiologists to make transfusions decisions and avoid unnecessary or delayed transfusions. Although several devices, such as satellite CO-Oximeters or point-of-care hemoglobinometers, can provide bedside Hb measurements, they are still invasive, time-consuming, and allow only intermittent monitoring [1–3]. Pulse CO-Oximetry (SpHb) (Masimo Corp., Irvine, CA, USA), based on near-infrared spectroscopy (NIRS) technology to detect microvascular light absorption of fingers, is currently available in providing non-invasive and continuous Hb monitoring in clinical scenarios [1, 4]. However, the light absorption signal derived from the arteriolar bed of peripheral tissues is strongly influenced by poor perfusion, such as hypothermia, hypovolemia, and vasoconstriction. Therefore, the signal source may be the main cause limiting the application of SpHb monitoring in severe bleeding, critically ill or shock patients [5, 6]. Previous studies have shown that light absorption signal from central large vessels, even from the ventricle, can be detected by placing sensors in the esophagus or trachea [7–9]. And the light absorption signal from central large vessel is more stable than that from peripheral microcirculation in critical patients [10]. When the light absorption signal is electronically amplified and recorded as a voltage signal, it is called photoplethysmography (PPG). The PPG signal contains an alternating current (AC) component caused by pulsatile arterial blood and a direct current (DC) component induced by non-pulsatile arterial blood and venous blood. Our previous study found that the esophagus provides an optimal window for detecting central PPG signals due to the relatively long contiguous relationship between the esophagus and the descending aorta [11, 12]. Transesophageal PPG signal from the descending aorta (dPPG) can be easily detected at an esophageal depth of 34.3 ± 1.4 cm at an orientation of 3 o’clock [11]. Therefore, PPG signal from the descending aorta merits further investigation as a potential alternative signal source for intraoperative continuous Hb monitoring. This pilot study was designed to test the feasibility and accuracy of noninvasive and continuous Hb monitoring using transesophageal dPPG signal in porcine model.

## Materials and methods

All the protocols were reviewed and approved by the Animal Ethics Committee of West China Hospital of Sichuan University (No. 2,019,056 A, Chengdu, China) and complied with the Animal Research Reporting In vivo Experiments (ARRIVE) guidelines.

### Transesophageal PPG sensor

**Fig. 1:**
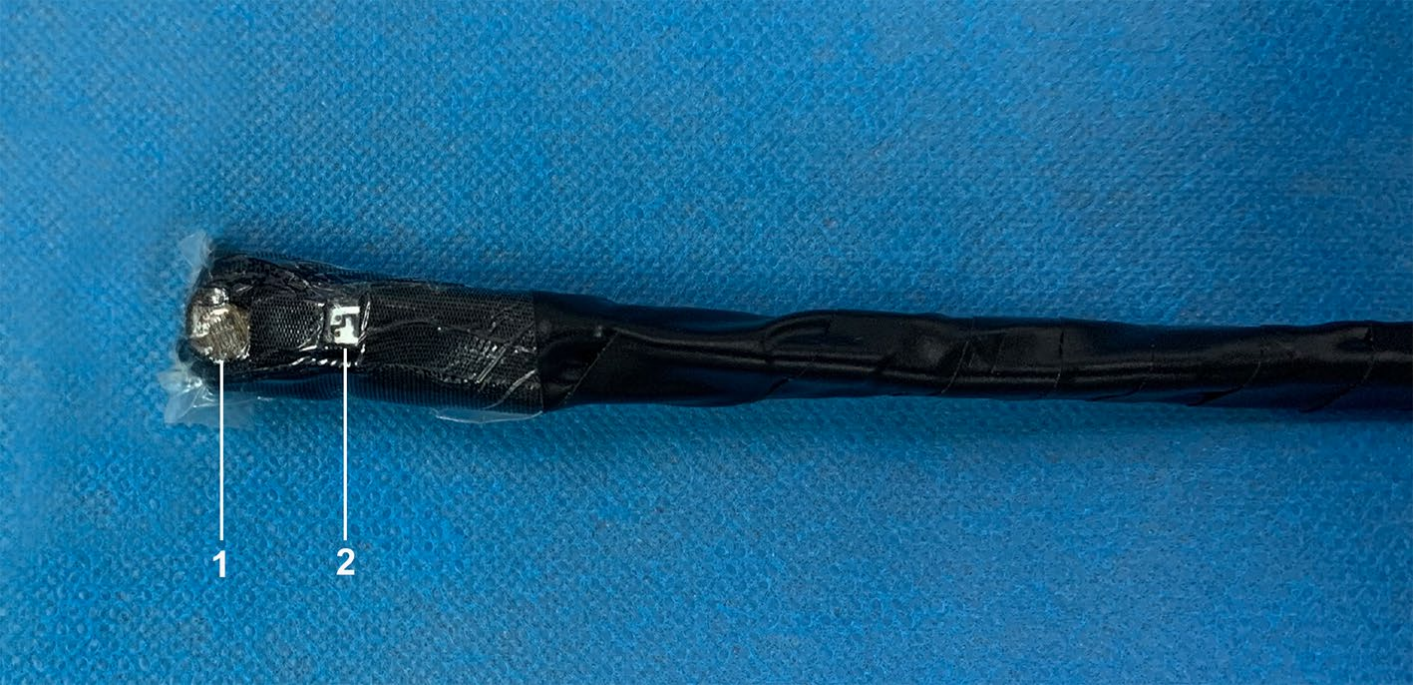
The modified transesophageal oximetry sensor. *1* detector of the oximetry sensor, *2* emitter of the oximetry sensor

A single-used oximetry sensor (Nellcor Puritan Bennett Inc., Pleasanton, CA, USA) was modified as a reflectance oximeter by shortening the distance between the emitters and photodetectors to 10 mm. The emitters contained 660 nm red light and 940 nm infrared light. The self-made sensor was fixed to a flexible string using a monolayer adhesive dressing (3 M, Shanghai, China) and black electrical tape (Fig. 1).

### Anesthesia

Landrace pigs aged 3–5 months and weighing 30–50 kg were anesthetized. After intramuscular injection of 3–5 mg/kg Zoletil 50 (tiletamine-zolazepam, Virbac, Carros cedex, France), a 14-gauge catheter was placed into the auricular vein. General anesthesia was induced with midazolam (0.04–0.08 mg/kg), sufentanyl (1–2 µg/kg), vecuronium (0.6-1 mg/kg) and maintained with sevoflurane inhalation (2–3%) and intermittent infusion of sufentanyl and vecuronium. After tracheal intubation, animals were mechanically ventilated with 100% fraction of inspired oxygen (FiO_2_). Mechanical ventilation parameters were necessarily adjusted to maintain the end-tidal carbon dioxide (EtCO_2_) in the normal range (35–45 mmHg). A 4 F catheter (PV2014L 16-A, Pulsion Pacific, Brisbane) was inserted into the femoral artery for continuous blood pressure monitoring, blood sampling for frequent Hb concentration measurement (Hb_i-STAT_) (i-STAT300, Attott Point of Care, Inc., Princeton, NJ, USA), and continuous cardiac output monitoring (PiCCO) (Pulsion Medical system SE, Feldkirchen, Germany). An 8.5 F, 3-lumen catheter (Yixinda, Shenzhen, China) was introduced into the internal jugular vein for central venous pressure (CVP) monitoring, removal of blood to induce hemodilution, and transfusion. The modified oximetry sensor was inserted into the esophagus and connected to a customized PPG acquisition and processing system (APS) [12]. Animals were kept on a warming pad to maintain nasopharyngeal temperature at 36 to 38 °C. Tail pulse oxygen saturation (SpO_2_), electrocardiogram (ECG), and nasopharyngeal temperature were continuously monitored.

### Acquisition of PPG signal, Hb measurement, and vital sign

The PPG waveform from the descending aorta has a slight dichrotic notch, which is completely different from the PPG signal from the aortic arch or left ventricle [11, 12]. Thus, the dPPG signal can be easily identified based on waveform characteristics. According to the PPG waveform displayed on the APS, the oximetry sensor was adjusted until a characteristic dPPG signal could be detected. The dPPG signals were separated into two channels (red and infrared) by a demultiplexer. The depth and orientation of the oximetry sensor in the esophagus were then fixed. If the dPPG waveform was unstable during the measurement, the sensor was slightly adjusted until the characteristic dPPG signal was displayed again on the APS. The dPPG signal was continuously recorded (16-bits resolution, 500-Hz sampling frequency) by the APS system and saved as a ‘txt’ file in a personal computer (hp pavilion dv2500). When the dPPG signal and hemodynamics were stable, a total of approximately 400–800 ml of blood was slowly withdrawn through the internal jugular vein catheter. Simultaneously, aliquots of crystalloid or colloidal solution were administered for hemodilution. The initially removed blood was stored in a disposable blood storage bag (Jie Rui, Shandong, China) containing sodium citrate. The Hb_i-STAT_ was measured after each 50 ml of blood removal and administration of aliquots of crystal or colloidal solution. Simultaneously, the corresponding 2-minute dPPG signal was collected and saved to pair the Hb_i-STAT_ measurement. The blood gas analysis device was calibrated according to manufacturer’s specifications before each experiment. The process of hemodilution was stopped when the Hb_i-STAT_ was < 60 g/L. The initially removed blood was then reinfused to increase the Hb concentration. Hb_i-STAT_ and the paired 2-minute dPPG signal were obtained after each 50 ml blood transfusion. BP, HR, SpO_2_ were recorded during the process of hemodilution and transfusion.

The saved dPPG signals, including red and infrared, were redisplayed as waveforms by a virtual instrument implemented in Matlab (R2007b; MathWorks, Inc., Natick, MA, USA) on the personal computer. Artifacts induced by the respiratory motion and esophageal peristalsis were removed by a filter (FIR, bandpass, 20th order). Metrics including AC component of red (AC_red_) and infrared (AC_inf_) light, DC component of red (DC_red_) and infrared (DC_inf_) light, AC_red_/DC_red_, and AC_inf_/DC_inf_ were extracted from dPPG [12]. All metrics were averaged from nine stable continuous waveforms in each 2-min segment of the dPPG signal. After the experiment, the animals were killed by injecting 10% potassium chloride.

### Statistical analysis and sample size

Continuous variables were tested for normal distribution using the Kolmogorov-Smirnov test and are presented as mean, standard deviation (SD), minimum and maximum. Pearson correlation coefficients were used to analyze correlations between the Hb_i-STAT_ and dPPG metrics. Linear regression analysis was performed to construct a model for Hb measurement by dPPG metrics (Hb_dPPG_). A receiver operating characteristic (ROC) curve was built to evaluate the performance of dPPG metrics in predicting the Hb “trigger threshold” for transfusion (Hb < 60 g/L and Hb > 100 g/L). The agreement between Hb_dPPG_ and Hb_i-STAT_ was demonstrated by a repeatedly measured Bland-Altman graph. Trending ability of Hb_dPPG_ to follow Hb_i-STAT_ changes was described by the polar plot. Paired Hb measured by dPPG and blood gas analysis was also plotted using the three zones Hb error grid analysis by Morey and colleagues, which takes into account the clinical significance of the difference [13]. Statistical analysis was performed using SPSS version 17.0 (SPSS, Inc., Chicago, IL, USA), SigmaPlot 12.0 (Systat Software, Inc., San José, CA, USA) and Matlab (R2007b; MathWorks, Inc., Natick, MA, USA). A P value of < 0.05 was considered statistically significant.

According to the standard error of the 95% limit of agreement (95% CI) is approximately $\sqrt{\text{3}{\text{s}}^{\text{2}}{\text{n}}^{\text{-1}}}$, where s is the SD of the differences between measurements by the two methods and n is the sample size. Expecting a SD of the differences between measurements by two methods of 20 g/L, a sample size of 300 measurements gives a 95% CI of about 0.22 s. Considering that at least sixteen paired measurements were performed per pig, nineteen pigs should be included.

## Result

### Demographics

The dPPG signal was successfully detected in all of the enrolled experimental pigs. No continuous 2-minute loss of dPPG signal occurred throughout the hemodilution and transfusion process. A total of 376 pairs of Hb_i-STAT_ and corresponding characteristic dPPG signal were obtained from nineteen pigs (33.0 ± 3.5 kg). The values of dPPG metrics and hemodynamic parameters during the measurements are listed in Table 1. The average decrease of Hb_i-STAT_ during the hemodilution procedure was 28.29 ± 10.69 g/L, and the average increase of Hb_i-STAT_ during transfusion was 23.38 ± 13.94 g/L. No esophagus bleeding was observed in any of the experiment animals after removal of the dPPG sensor.

**Table 1:** Values of dPPG metrics and hemodynamic parameters during the measurement

Parameters	Mean	SD	Minimum	Maximum
AC_red_ (v)	0.19	0.15	0.01	0.75
DC_red_ (v)	6.90	5.24	0.27	17.71
AC_red_/DC_red_ (%)	3.13	1.35	1.13	6.72
AC_inf_ (v)	0.11	0.13	0.01	0.71
DC_inf_ (v)	3.13	3.03	0.01	0.83
AC_inf_/DC_inf_ (%)	3.73	1.45	0.95	7.07
Hb_i−STAT_ (g/L)	83.78	16.55	51	143
CO (L/min)	3.87	1.02	1.84	6.79
SBP (mmHg)	119.68	25.77	68	174
DBP (mmHg)	69.73	20.14	34	119
MAP (mmHg)	95.49	27.14	45	165
HR (bpm)	94.77	19.17	56	159
SpO_2_ (%)	99.82	0.47	98	100

*Abbreviations*: dPPG, descending aortic photoplethysmography; AC_red_, alternating current of red light; DC_red_, direct current of red light; AC_inf_, alternating current of infrared light; DC_inf_, direct current of infrared light; v, voltage; Hb_i−STAT_, hemoglobin measured by i-STAT device; CO, cardiac output; SBP, systolic blood pressure; DBP, diastolic blood pressure; MAP, mean arterial pressure; SpO_2_, pulse oxygen saturation

### Assessment of correlation

**Fig. 2:**
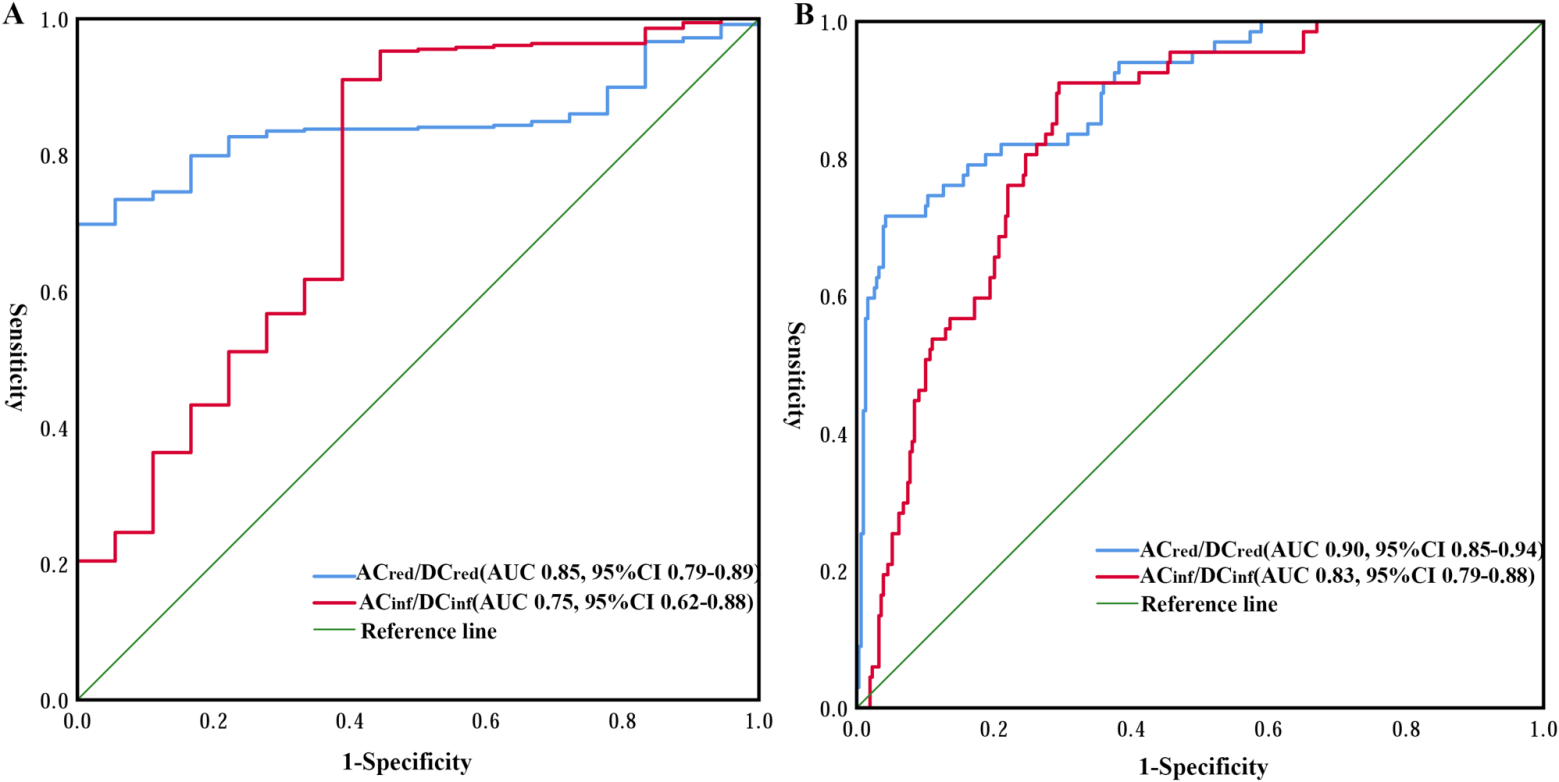
Showed the ROC curves for AC_red_/DC_red_ and AC_inf_/DC_inf_ in predicting Hb < 60 g/L (**A**) and Hb > 100 g/L (**B**). ROC, receiver operating characteristic curve; AC_red_, alternating current of red light; DC_red_, direct current of red light; AC_inf_, alternating current of infrared light; Hb, hemoglobin concentration

The dPPG metrics including AC_red_, DC_red_, and DC_inf_ had mild correlations with Hb_i-STAT_, with correlation coefficients of 0.102 (*p* = 0.047), -0.221 (*p* < 0.001), and − 0.243 (*p* < 0.001), respectively. AC_red_/DC_red_ (*r* = 0.790, *p* < 0.001) and AC_inf_/DC_inf_ (*r* = 0.782, *p* < 0.001) exhibited moderate correlations with Hb_i-STAT_. No significant correlation was found between AC_inf_ and Hb_i-STAT_ (*r* = 0.064, *p* = 0.218). ROC curve analysis identified an AC_red_/DC_red_ value of 2.37% and an AC_inf_/DC_inf_ value of 2.06% as the best cut-off point in predicting Hb_i-STAT_ < 60 g/L (Fig. 2A). The areas under the ROC curve (AUC) of AC_red_/DC_red_ and AC_inf_/DC_inf_ were 0.85 (95% CI 0.79–0.89) and 0.75 (95% CI 0.62–0.88), respectively. The AUC of AC_red_/DC_red_ and AC_inf_/DC_inf_ in predicting Hb_i-STAT_ > 100 g/L were 0.90 (95% CI 0.85–0.94) and 0.83 (95% CI 0.79–0.88), respectively, with cut-off values of 3.89% and 4.36% (Fig. 2B).

### Assessment of accuracy

In the case of intercorrelation, the dPPG metrics including AC_red_/DC_red_ and AC_inf_/DC_inf_ were incorporated into a stepwise multi-linear regression analysis to construct a mathematical model of Hb_dPPG_.

Hb_dPPG_ = 341.84 × AC_red_/DC_red_ + 569.61 × AC_inf/_DC_inf_ + 49.87.

**Fig. 3:**
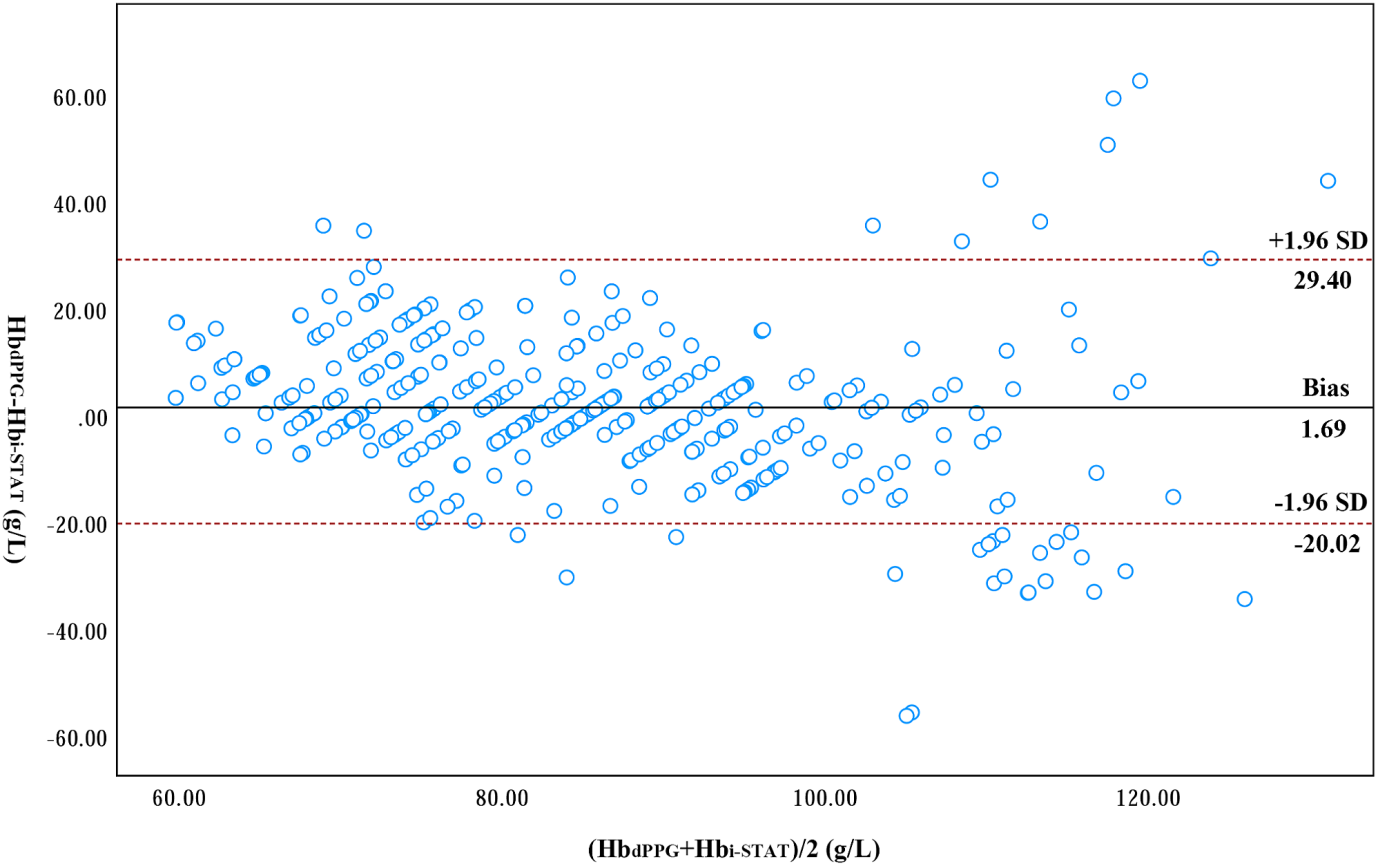
Bland-Altman plot showed the bias and limit of agreement between Hb measured by transesophageal dPPG signal and blood gas analysis dPPG, descending aortic photoplethysmography

**Fig. 4:**
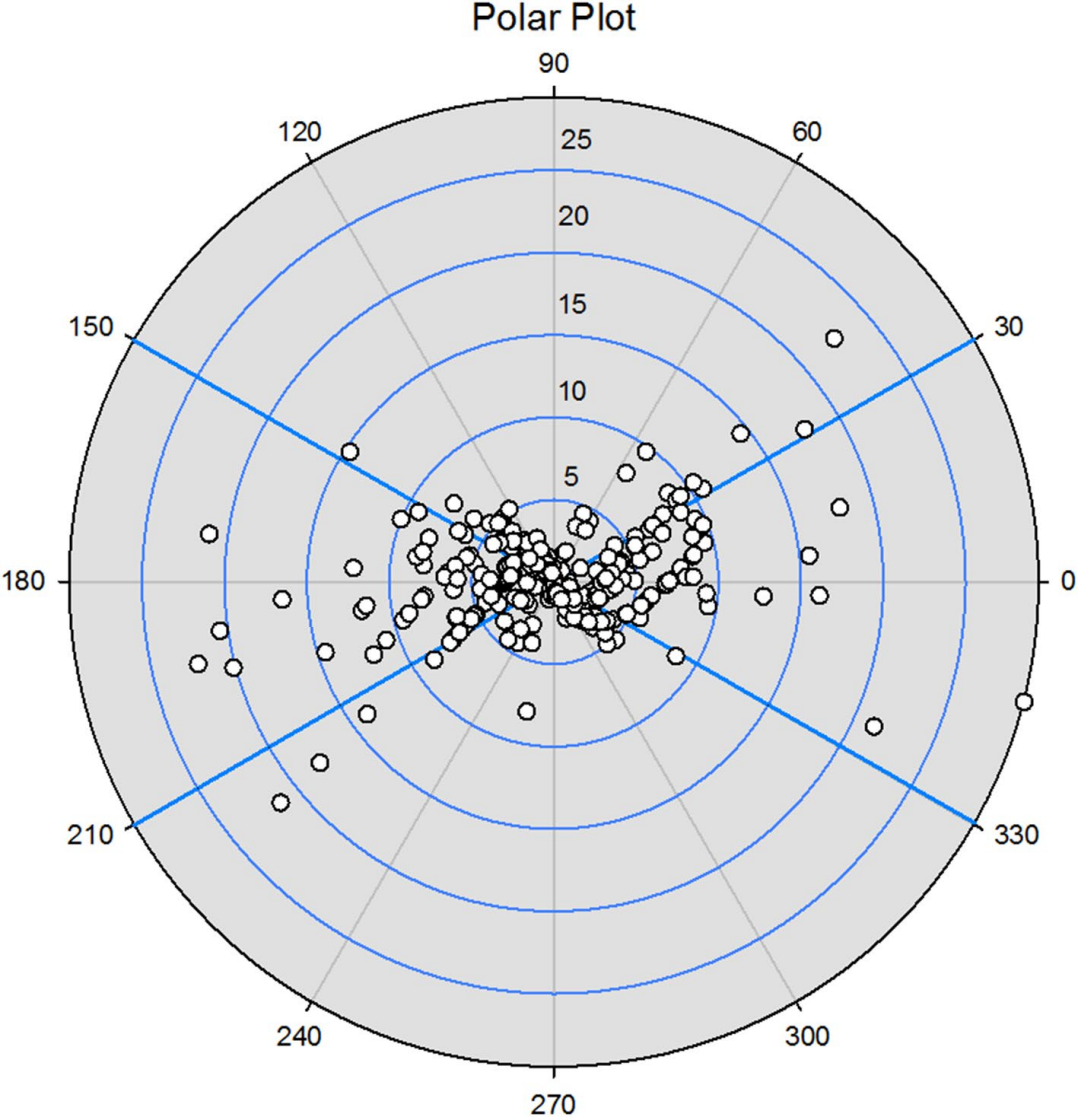
Polar plot analysis on trending ability of changes of Hb measured by transesophageal dPPG signal against the blood gas analysis

**Fig. 5:**
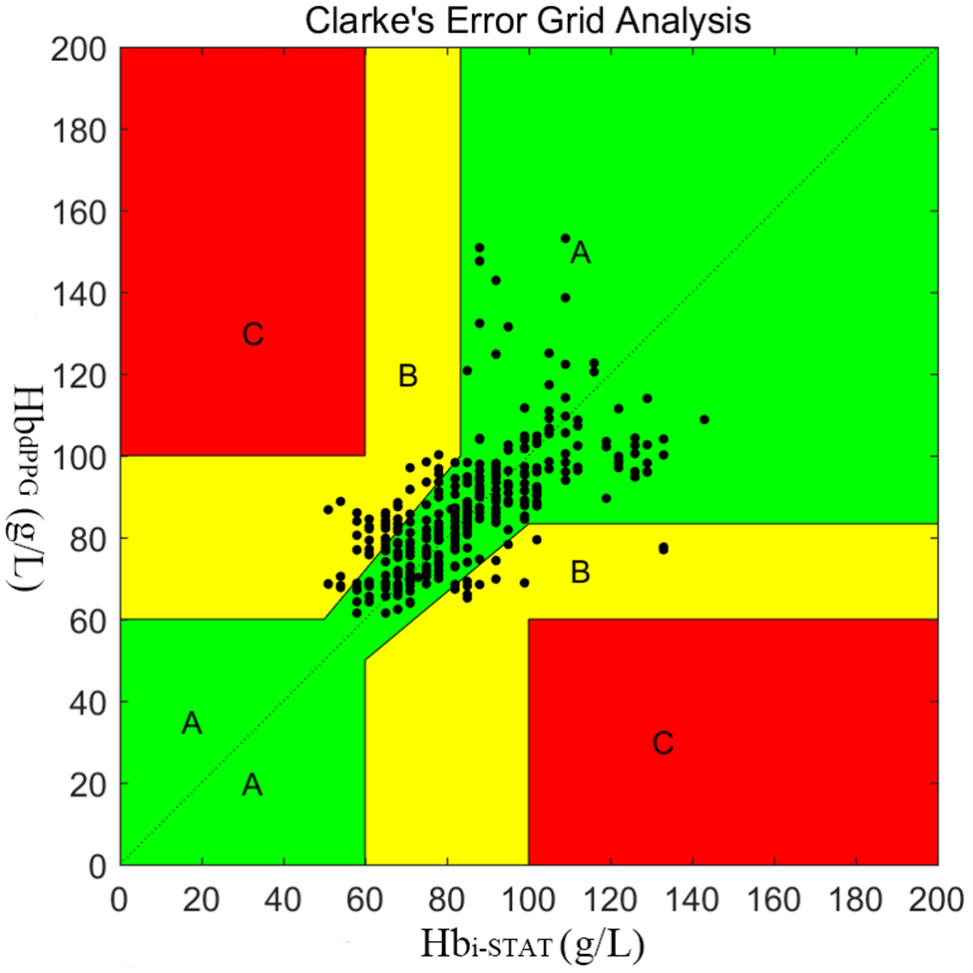
Paired Hb values provided by transesophageal dPPG and blood gas analysis plotted using the three zones Hb error grid taking into account the clinical significance of the difference

The correlation coefficient between Hb_dPPG_ and Hb_i-STAT_ was 0.81 (*p* < 0.001). A Bland-Altman analysis is shown in Fig. 3. The bias (precision) between Hb_dPPG_ and Hb_i-STAT_ was 1.69 g/L (14.13 g/L), and the limit of agreement (LOA) was − 26.02–29.40 g/L with a percentage error of 33%. In the trending analysis, the polar plot exhibited that the concordance rate between Hb_dPPG_ and Hb_i-STAT_ was 65% (Fig. 4). Clinical significance analysis of the 376 discrete points is presented in Fig. 5. 82% (307/376) of the data lay within the Zone A, 18% (69/376) within the Zone B and 0% (0/376) within the Zone C.

## Discussion

In this pilot study, we demonstrated the feasibility of noninvasive and continuous hemoglobin monitoring by transesophageal dPPG signal in porcine hemodilution and transfusion model. The dPPG metrics, including AC_red_/DC_red_ and AC_inf/_DC_inf_, had significant correlations with Hb_i−STAT_, and AC_red_/DC_red_ enabled a sensitive prediction of Hb_i−STAT_ < 60 g/L and Hb_i−STAT_ > 100 g/L. In addition, we found that Hb_dPPG_ was moderately correlated with Hb_i−STAT_.

The SpHb obtained by a transcutaneous spectrophotometry-based technology suggests that the Hb information can be extracted from NIRS signal. It has the advantage of continuity and noninvasiveness of online assessment of hemoglobin concentration, and significantly reduces the need for blood transfusion and costs [14]. Although the accuracy and trending ability of SpHb have been well documented in stable hemodynamic or in slow and of small magnitude hemoglobin changes, it’s inaccuracy of Hb assessment in critically ill patients has been described [15–18]. In severely traumatized and surgical bleeding patients, SpHb showed decreased evaluate ability and a high detection failure rate [6]. In acute hemorrhagic patients, SpHb showed significantly lower readings than the laboratory measurement to assess Hb concentration [19, 20]. Park YH et al. reported that the bias between SpHb and laboratory measured Hb increased with poor peripheral perfusion [21, 22]. These results suggest that the limited application of SpHb in critically ill patients may be mainly due to the susceptibility of peripheral spectroscopy signal to impaired tissue perfusion, hypotension, hypothermia, and infusion of vasopressor [1, 5, 23]. This suggests that using a stronger and more stable NIRS signal source to monitor Hb merits investigation. Therefore, in our study, we used central dPPG as an alternative signal source for continuous hemoglobin monitoring.

To our knowledge, this is the first study to use central rather than the peripheral PPG signal for Hb measurement. Our study found that several metrics extracted from the dPPG signal, especially AC_red_/DC_red_ and AC_inf_/DC_inf_, have significant correlations with Hb_i−STAT_, which was consistent with a previous study using finger PPG signal in healthy subjects [24]. ROC analysis showed the ability of AC_red_/DC_red_ to reliably predict the Hb “trigger threshold” for transfusion (Hb_i−STAT_ < 60 g/L and Hb_i−STAT_ > 100 g/L) (Fig. 2). These results suggest that it was feasible to extract information about Hb concentration from the dPPG signal.

The bias (precision) of SpHb ranged from − 0.20 g/dL (-2.0 g/L) to 1.63 g/dL (16.3 g/L) (1.0–2.7 g/dL or 10.0–27.0 g/L) in spine surgery, cardiac surgery, and acute gastrointestinal bleeding patients [1]. The bias of Hb_dPPG_ measurement in our study was similar to that reported in the literature for SpHb assessment in a mixed surgical population [19, 25]. The correlation coefficient (*r* = 0.81) between Hb_dPPG_ and Hb_i−STAT_ was also consistent with the correlation between SpHb and laboratory Hb measurement in surgical patients [19]. Transesophageal dPPG measures the light absorption of the macrovascular blood flow, the descending aorta, rather than the microvascular net. On the one hand, the central macrovascular dPPG signal carries more information about Hb than the peripheral microvascular PPG signal. On the other hand, the signal intensity of central reflection dPPG signal (signal-to-noise ratio: 0.03–0.04) is much greater than peripheral reflection PPG signal (signal-to-noise ratio: 0.001–0.005) [11, 26]. In our study, dPPG signals were still detected even during the periods of low cardiac output or hypotension, whereas the tail SpO_2_ waveform was occasionally became disturbed or even disappeared. In some specific pathological circumstances, such as vasoplegia syndrome, peripheral arterial tension is reduced, leading to a significantly reduction in the pulsatile component of the PPG signal, whereas the central descending aorta is less affected. Therefore, the PPG that detected from the central large vessel was more stable than the peripheral PPG signal. The central dPPG technology may avoid erroneous Hb assessment and transfusion decisions in acute hemorrhage, hypothermia, vasopressors administration, and vasoplegia syndrome.

However, there was a generally wide LOA and a large percentage error between Hb_dPPG_ and Hb_i−STAT_. And the Hb_dPPG_ also showed poor comparability with the Hb_i−STAT_ measured of hemoglobin change trends during the process of hemodilution and transfusion. These results may be associate with several factors. First, although the artifacts caused by respiratory motion and esophageal peristalsis hidden in the transesophageal dPPG signal were removed by a filter, the nonpulsatile DC component had contamination of background light absorption caused by surrounding tissues, such as the esophageal mucosa and smooth muscle between the transesophageal oximetry sensor and the descending aorta [12, 27, 28]. Similar to the principle of cerebral oxygen saturation monitoring, it may be possible to remove background light absorption contamination by setting both near and far photodetectors or adding different wavelengths related to Hb light absorption, but these require further investigation [22, 29, 30]. In addition, the infusion of colloids appears to affect the light absorption of hemoglobin [4]. In our study, colloids and crystalloids were frequently infused during the hemodilution process, making it difficult to evaluate the effect of fluids on Hb_dPPG_ measurement. Third, a recent pilot study found that the light absorption of hemoglobin was influenced by high concentrations of oxygen inhalation [31]. In our study, the experimental animals were ventilated with pure oxygen, so we cannot evaluate the influence of FiO_2_ on Hb_dPPG_ measurement. Finally, the large percentage error of Hb_dPPG_ may also be related to the reference equipment for Hb standard values in this study. The measurement of Hb_i−STAT_ is not the gold standard and is strongly influenced by the increase in white blood cells, high blood lipids, low total protein, and low hemoglobin [32]. Although Hb_dPPG_ has poor trending ability, it is still considered by the authors as a good device for anesthesiologists to continuously detect the trend of Hb concentration change. It could facilitate timely reactions of anesthesiologists to treat potential decrease of Hb. Worthy of note, the transesophageal dPPG monitoring in our study provided 100% continuous signal during the process of hemodilution and transfusion, even though in the presence of low cardiac output and low blood pressure. Therefore, timely establishment of continuous Hb_dPPG_ measurements by transesophageal dPPG monitoring could guide transfusion and resuscitation in critical patients. Based on current technology, introducing standard values of Hb for correction or data accumulation is worth exploring to further improve the accuracy and trending ability of Hb_dPPG_ measurement.

The error grid allows assessment of inaccuracy of devices according to their impact on patient care [13]. Morey et al. suggested that an ideal device should have 95% of points within the zone A, 5% within the zone B and 0% within the zone C. Although the Hb_dPPG_ measurement does not meet the proposed criteria (82/18/0% in our study), no data points correlated with potential therapeutic misadventure according to the 2006 transfusion criteria of American Society of Anesthesiologists [33].

For continuous transesophageal Hb_dPPG_ monitoring, safety should be considered. In our study, no esophageal bleeding was observed after removal of the dPPG sensor in any of the experimental animals. In our previous clinical studies, there was no complaint of sore throat, dysphagia or dysarthria in all enrolled patients after insertion of the oximetry sensor into esophagus [11, 12]. In order to reduce the potential damage caused by placing the dPPG sensor into the esophagus, the dPPG sensor could be assembled into a tubule. Thus, it can be easily inserted into the esophagus with minimal invasiveness like placing a stomach tube. However, the safety of the dPPG sensor still needs to be validated in further studies.

This study has several limitations. First, although 376 pairs of valid data were analyzed, the sample size of this study was small. Second, the calculation model of Hb_dPPG_ has not been validated in a new animal or clinical experiment by online display of Hb_dPPG_ values. Third, we used Hb_i−STAT_ as the reference method in this study, which is not the true gold standard of Hb measurement. Fourth, we did not collect peripheral PPG signal to compare with the central dPPG signal, nor did we collect peripheral SpHb assessment to compare with central Hb_dPPG_. Fifth, the lowest observed Hb_i−STAT_ level was 51 g/L and the lowest observed Hb_dPPG_ level was 61 g/L. Future work should elucidate the ability of transesophageal Hb_dPPG_ monitoring to detect Hb concentrations over a wide range. Finally, the sensor used in our study was self-made, with the light emitter and photodetector aligned side-by-side. Although we did not observe any unexpected adverse events or effects in our study, some precautionary aspects, such as the safety of the esophageal mucosa and the heart when electrocautery or defibrillation is used, are still unknown. Therefore, it has not yet been tested in patients before developing a sensor specifically for transesophageal Hb_dPPG_ monitoring.

## Conclusion

In conclusion, this study demonstrated that it is feasible to establish a noninvasive and continuous Hb monitoring by transesophageal descending aortic PPG signal when the oximetry sensor is positioned properly. The AC_red_/DC_red_ extracted from the dPPG signal could provide a sensitive prediction of the Hb threshold for transfusion. The Hb concentration measured by dPPG signal has moderate correlation with that measured by blood gas analysis. Transesophageal Hb_dPPG_ monitoring merits further evaluation and development for high risk of surgical bleeding patients or critically ill patients in intensive care units, or operating rooms.

## Data Availability

The datasets used and analysed during this study are available from the corresponding author on reasonable request.

## Abbreviations

Hb: hemoglobin
dPPG: descending aortic photoplethysmography
NIRS: near-infrared spectroscopy
AC: alternating current
DC: direct current
ROC: receiver operating characteristic
Hb_dPPG_: hemoglobin measured by descending aortic photoplethysmography
Hb_i−STAT_: hemoglobin measured by blood gas analysis
AC_red_: alternating current of 660 nm red light
DC_red_: direct current of 660 nm red light
AC_inf_: alternating current of 940 nm infrared light
DC_inf_: direct current of 940 nm infrared light
SpHb: Pulse CO-Oximeter
PPG: photoplethysmography
FiO_2_: fraction of inspired oxygen
EtCO_2_: end-tidal carbon dioxide
PiCCO: continuous cardiac output monitoring
CVP: central venous pressure
APS: acquisition and processing system
SpO_2_: pulse oxygen saturation
ECG: electrocardiogram
BP: blood pressure
HR: heart rate
SD: standard deviation
CI: confidence interval
LOA: limit of agreement
